# Dopaminergic Vulnerability and Motor Impairments in Older Adults: Need for Targeted Pharmacotherapy

**DOI:** 10.1093/geroni/igaa057.2730

**Published:** 2020-12-16

**Authors:** Nicolaas Bohnen

**Affiliations:** University of Michigan, Ann Arbor, Michigan, United States

## Abstract

Motor impairments, including slow walking, are common in older age in the absence of prototypical Parkinson’s disease (PD). The etiology of such disturbances is heterogeneous and multi-system in nature. Dopamine is a key neurotransmitter involving motor, cognitive and behavioral circuitry. Normal aging is associated with substantial dopaminergic losses in the brain. Dopaminergic vulnerability of aging can be augmented by genotypic changes that may further compromise dopaminergic signaling, such as polymorphisms in the COMT gene. These observations may augur dopaminergic pharmacotherapy studies to treat gait disturbances in older adults. Prior dopaminergic therapy studies have shown overall limited effects in non-PD older adults. A major limitation of these studies is the non-targeted selection calling for personalized medicine approaches. Recent dopaminergic treatment studies targeting specific sub-groups of non-PD older adults conducted by us and others will be discussed.

